# The roles of integrins in cancer

**DOI:** 10.12703/r/10-45

**Published:** 2021-05-07

**Authors:** Donatella Valdembri, Guido Serini

**Affiliations:** 1 Candiolo Cancer Institute - Fondazione del Piemonte per l’Oncologia (FPO) - IRCCS, Candiolo (TO), Italy; 2 Department of Oncology, University of Torino School of Medicine, Candiolo (TO), Italy

**Keywords:** Integrins, cancer

## Abstract

Integrin-mediated adhesion of cells to the extracellular matrix (ECM) is crucial for the physiological development and functioning of tissues but is pathologically disrupted in cancer. Indeed, abnormal regulation of integrin receptors and ECM ligands allows cancer cells to break down tissue borders, breach into blood and lymphatic vessels, and survive traveling in suspension through body fluids or residing in metabolically or pharmacologically hostile environments. Different molecular and cellular mechanisms responsible for the modulation of integrin adhesive function or mechanochemical signaling are altered and participate in cancer. Cancer development and progression are also bolstered by dysfunctionalities of integrin-mediated ECM adhesion occurring both in tumor cells and in elements of the surrounding tumor microenvironment, such as vascular cells, cancer-associated fibroblasts, and immune cells. Mounting evidence suggests that integrin inhibitors may be effectively exploited to overcome resistance to standard-of-care anti-cancer therapies.

## Introduction

In multicellular organisms, the interaction of cells with the surrounding extracellular matrix (ECM) is crucial for the physiology and integrity of tissues. In the mammalian cell, integrins are the main ECM receptors providing a link between the external matrix scaffold and the internal actin cytoskeleton mediated mainly by their cytosolic adaptor proteins, among which talin and kindlin play pivotal roles^1,2^.

The 18 α integrin and eight β integrin distinct subunits are assembled in various combinations and form 24 transmembrane heterodimers that are endowed with binding specificity for different ligands^3,4^. Integrin receptors are regulated at transcriptional and translational levels in a tissue-specific manner. Moreover, endosomal trafficking back and forth from the plasma membrane, surface-polarized distribution, and dynamic localization at ECM contact sites are all crucial regulatory steps of integrin-mediated adhesion. Integrin functions are disrupted in many pathological settings, such as cancer. Indeed, alterations in cell adhesion and migration are involved in almost every phase of cancer progression, from tumor onset to metastasis. Moreover, abnormal expression of integrins has been correlated to several types of cancer and varies depending on the extent of neoplastic progression and prognosis. In addition, altered endocytosis and recycling kinetics, often observed in cancer cells, result in derailed cell surface and endosomal integrin signaling, thus providing cancer cells with oncogenic and invasive abilities^3,4^. Finally, although increasing evidence points to integrins as potential therapeutic targets in several cancer types, clinical trials so far have achieved poor outcomes^3,4^.

To develop new and effective pharmacological anti-cancer strategies, investigators need to thoroughly understand the contribution of integrin activation and signaling to the different steps of tumor progression. Recent reviews^3,4^ have analyzed in detail the functions and roles that integrins play in cancer development, progression, and treatment. Here, we will focus mainly on the most recent advances that have further unveiled the molecular and cellular mechanisms by which integrins affect the biology and therapy of cancer.

## Regulation of integrin function and signaling in cancer cells

### Integrin conformational activation

**Figure 1.**
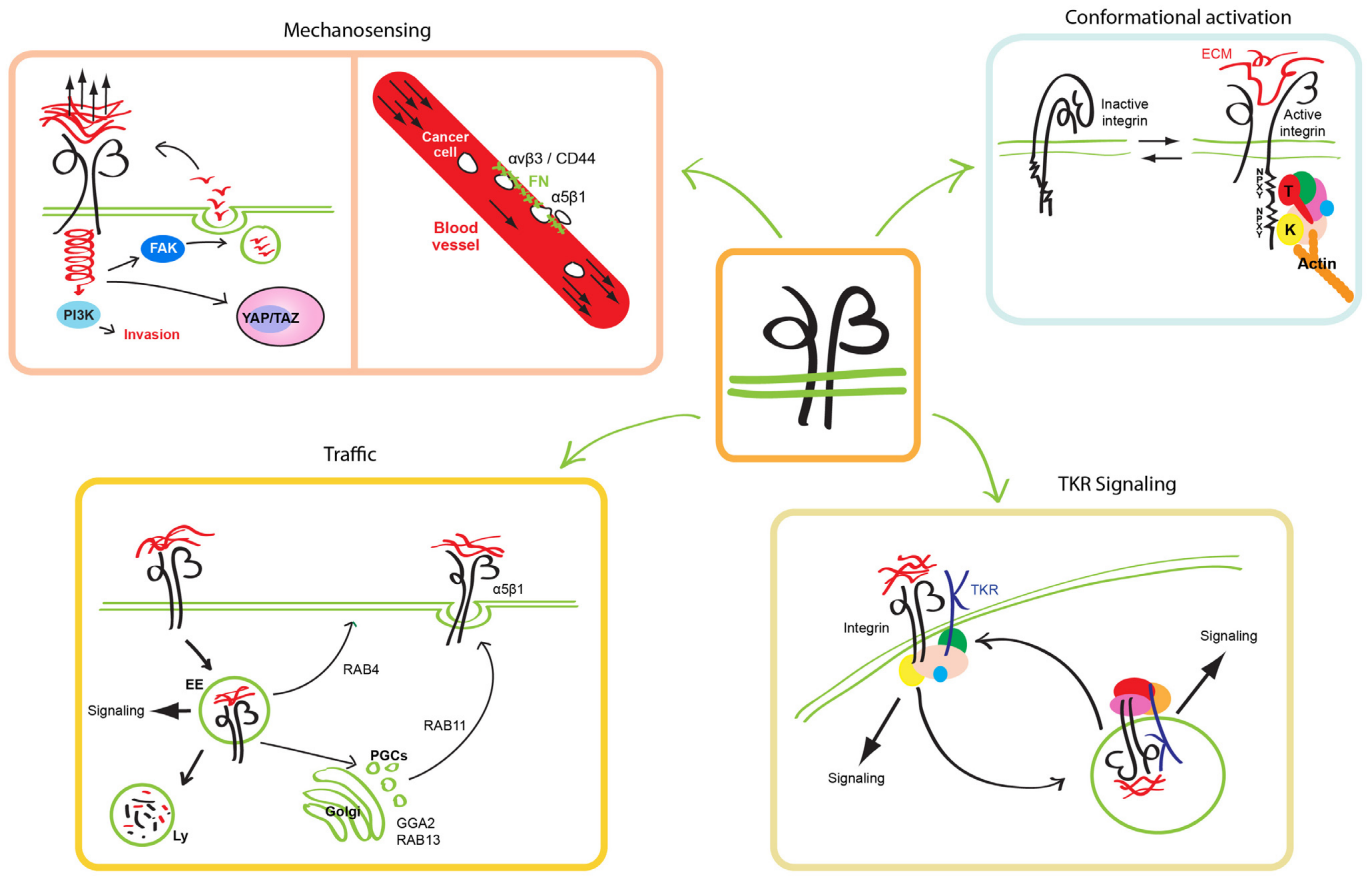
Integrin function is regulated by conformational activation and vesicular traffic and is crucially involved in mechanosensing and TKR signaling. Integrin αβ heterodimers shift between a low-affinity bent-closed (inactive) conformation and a high-affinity extended-open (active) conformation. Many interactors (collectively identified as “the adhesome”) bind the cytoplasmic portion of conformationally active integrins; among them, talin (T) and kindlin (K) are the only two β subunit adaptor proteins found to be indispensable for integrin activation (*upper right panel*). Integrins sense the biophysical properties of the surrounding microenvironment. Stiff substrates promote an increased force transmission and YAP/TAZ translocation to the nucleus, whose target genes promote cancer progression. In blood vessels, low hemodynamic forces promote the binding of circulating tumor cells to fibronectin (FN) secreted by vascular endothelial cells; in this process, αvβ3 integrin/CD44 first and α5β1 integrin later promote cancer cell arrest. Stiff extracellular matrix (ECM) sustains PI3K and FAK signaling (*upper left panel*). Integrins are constitutively endocytosed and recycled back to the cell surface in a short loop (RAB4-dependent) or long loop (TGN and RAB11-dependent) or sorted for degradation in lysosomes (Ly). The escape of integrins from the degradative pathway sustains their signaling from endosomes (EE) and promotes their recycling to the surface (*lower left panel*). Mutual regulation between integrin and growth factor TKR signaling and traffic is crucial for cancer development and metastatic dissemination (*lower right panel*). TKR, tyrosine kinase receptor; PGC, post-Golgi carrier.

Integrins fluctuate between a low-affinity bent-closed conformation and a high-affinity extended-open conformation, the shift being defined as integrin activation. Despite the large number of integrin interactors, which constitute the so-called “adhesome”, talin and kindlin are the only two integrin-binding adaptor proteins known to be indispensable for integrin activation^5^. Talin recruitment to the membrane-proximal NPXY motif of the β integrin cytoplasmic tail disrupts the transmembrane association between integrin α and β subunits, inducing the extension of the extracellular portion and the opening of the ligand-binding pocket^1^. Kindlin family members (kindlin-1, -2, and -3) assist talin-dependent stabilization of the extended-open conformation of integrins and their clustering. Binding the cytosolic membrane-distal NPXY motif of integrin β subunits, kindlins function as protein–protein interaction hubs capable of recruiting molecular complexes and allowing integrin connection to the actin cytoskeleton and cell spreading (Figure 1)^2^.

All three kindlin isoforms have been involved in cancers, as their expression levels have been found to be altered compared with the corresponding normal tissue in several cancer types. Kindlin-1 is frequently overexpressed in lung, breast, bladder, and colon cancer^6^ and its oncogenic potential strongly depends on transforming growth factor beta (TGFβ) signaling. Kindlin-2, the most broadly distributed member of the family, exerts a tumor type–dependent promoting or inhibiting function. In breast^7^ and bladder^8^ cancer, pancreatic ductal adenocarcinoma (PDAC)^9^, and malignant mesothelioma, kindlin-2 expression directly correlates with tumor progression and invasion^10^. Most likely, kindlin-2 exerts its oncogenic function in these tumors by promoting epithelial-to-mesenchymal transition (EMT), fostering ERK and Akt activation, and TGFβ signaling. In contrast, in colorectal carcinomas and serous epithelial ovarian cancers, kindlin-2 suppresses tumor invasion and progression^11,12^. Interestingly, kindlin-2 displays integrin-dependent and -independent roles in cancer development. In esophageal squamous cell carcinoma cells, kindlin-2/β1 integrin/AKT signaling activation promotes tumor invasion^13^; in prostate cancer cell lines, inhibition of the kindlin-2/β1 integrin pathway enhances the sensitivity to chemotherapeutic agents^14^. In contrast, in breast cancer cells, TGFβ signaling increases the kindlin-2–mediated but integrin-independent expression of colony-stimulating factor 1 (CSF-1) that recruits macrophages, driving tumor progression^15^. Moreover, by binding nuclear p53 and impairing the transcription of the senescence inducers SerpinB2 and p21, kindlin-2 promotes breast cancer cell growth^16^. Kindlin-3, which is expressed mainly in the hematopoietic system, has been found to support the progression of acute myeloid leukemia and chronic myeloid leukemia (CML)^17^. In a CML mouse model, the loss of kindlin-3 abrogates integrin-mediated adhesion of leukemia stem cells to the bone marrow niche, triggering their release into circulation and impairing their proliferation, survival, and metastatic dissemination^18^.

### Integrins and mechanosensing

Cells explore the biophysical properties of the surrounding microenvironment through integrin receptors and respond by activating intracellular signals and adapting their behavior accordingly. This process, termed mechanotransduction, is often dysregulated during cancer progression, which usually is characterized by increasing tissue stiffness due to abnormal ECM deposition, organization, and rehandling^19^. In particular, stiff substrates promote an increased force transmission that supports the reprogramming of oncogene-expressing cells toward a tumorigenic phenotype^20^. Stiff matrices induce integrin- and F-actin–dependent activation of the transcription factors YAP and TAZ, whose target genes account for a substantial fraction of the transcriptional responses downstream of oncogenic signaling^21^. Recently, tumor ECM meshworks enriched by agrin^22^ and periostin^23^ have been discovered to exploit integrin-activated signaling pathways that impinge on YAP/TAZ-driven gene transcription (Figure 1).

Mechanical properties of tumor ECM are also influenced by collagen fiber crosslinking that requires fibronectin (FN). ECM rich in collagen-FN favors exposure and ligation of the FN cell adhesion synergy site by α5β1 integrin and enhances zyxin and vinculin recruitment, finally sustaining PI3K-dependent tumor cell invasion^24^. Increased extracellular tension can also be due to the bulkiness of the cancer cell glycocalyx. In recurrent glioblastoma multiforme (GBM), high levels of glycoconjugates induce a mesenchymal stem–like phenotype and promote integrin adhesion assembly and FAK-mediated signaling, which in turn sustain the expression of several glycoproteins in a self-reinforcing mechanism^25^. Furthermore, mechanosensing is involved in cancer metastasis. Likely owing to conformational activation properties of adhesion receptors, such as integrin catch bonding behavior, pN-range blood flow hemodynamic forces promote the binding of circulating tumor cells to vascular endothelial cells (ECs)^26^. In particular, halting of circulating cancer cells occurs in vascular regions where lower shear forces favor the luminal deposition of FN; it is a two-step process, first mediated by CD44 and αvβ3 integrin weak adhesions and then stabilized by α5β1 integrin-dependent high-strength adhesion (Figure 1)^27^.

In addition to stiffness, the geometric physical features of ECM may influence tumor progression. For example, alterations of ECM architecture can promote vasculogenic mimicry, a cancer cell–based blood supply system that can take place independently of angiogenesis and is associated with poor prognosis. Collagen matrices with small pores and short fibers upregulate β1 integrin expression and increase motility and expression of vasculogenesis-associated genes in tumor cells^28^.

### Integrin traffic

Integrin traffic contributes to ECM adhesion turnover and ultimately regulates cell migration and invasion. According to the canonical model, integrins are constitutively endocytosed in a clathrin- or caveolin-dependent manner and are recycled back to the cell surface or sorted for degradation^29^. Integrin recycling to the cell surface follows two different routes: a short Rab4-mediated loop or a long Rab11-dependent loop^30^. The latter is the preferential pathway followed by active α5β1 integrins and is crucial for cancer cell migration and colonization of metastatic sites. In breast cancer cells, silencing of the Rab11 regulator Rabgap1 impairs active β1 integrin recycling and migration^31^, whereas Rab11b upregulation during early adaptation to the brain metastatic sites favors successful β1 integrin recycling to the surface and interaction with the ECM, promoting mechanotransduction-activated survival^32^. Rab11b-dependent active β1 integrin basolateral recycling exploits the trans-Golgi network (TGN) secretory pathway^33^, and GGA2 (Golgi-associated, gamma adaptin ear-containing, ARF-binding protein 2) and Rab13 are key specificity determinants of this route (Figure 1)^34^.

The escape of integrins from the degradative pathway sustains their endosomal signaling and promotes their recycling to the surface. In breast cancer cells, upon 17β-estradiol stimulation, FN-bound active β1 integrins are internalized together with estrogen receptor alpha (ERα) and recycled to the plasma membrane in a Rab11-dependent manner, thus preventing ERα lysosomal degradation^35^. Consistently, an FN-rich matrix induces tamoxifen resistance in breast cancer and correlates with lower patient survival^36^. In small-cell lung cancer, low expression levels of cullin 5 (CUL5) and suppressor of cytokine signaling 3 (SOCS3) correlate with a highly metastatic phenotype and poor prognosis. Mechanistically, CUL5 and SOCS3 deficiency impairs the formation of the cullin-RING E3 ubiquitin ligase complex, preventing β1 integrin degradation and stabilizing downstream FAK/SRC signaling^37^. Moreover, β1 integrin-promoted FAK signaling on endosomes^38,39^ and sustained β1 integrin co-internalized tyrosine kinase receptor (TKR) signaling^39^ suppress cell death due to ECM detachment (anoikis).

Intercellular communication through extracellular vesicle–dependent integrin-mediated endocytosis and recycling also plays a critical role in metastatic dissemination^40^. αvβ3 integrin is required to allow extracellular vesicle–dependent enhancement of colony-forming capacity in cultured breast cancer cells^41^. Whereas heparan sulfate proteoglycans act as receptors responsible for extracellular vesicle tethering and capture on the surface of breast cancer cells, αvβ3 integrin mediates the ensuing FAK- and dynamin-driven endocytosis^41^. PDAC cells carrying loss-of-function p53 mutation (mutp53) instead release exosomes that contain high levels of podocalixin and can make other organs more favorable to metastatic seeding^42^. Specifically, the uptake of mutp53 PDAC exosomes by otherwise-normal fibroblasts localized in distant districts from the site of origin, such as the lungs, promotes α5β1 integrin recycling and the deposition of a highly pro-invasive ECM^42^.

### Integrins and growth factor receptor signaling

It is well established that interconnections between integrin and growth factor TKR signaling and traffic are crucial for cancer development and metastatic dissemination (Figure 1)^43^. Among TKRs, ligand-activated epidermal growth factor receptor (EGFR) was previously reported to associate with several integrin subunits and act upstream to modulate their adhesive and signaling functions^44^. Recently, the functional meaning of previously reported^45^ non-canonical ligand-independent signaling of EGFR family members downstream of ECM-bound integrins was deciphered^46^. It turns out that, owing to transient interactions with integrins at peripheral nascent adhesion sites of spreading cells, EGFR and human epidermal growth factor receptor 2 (HER2) get activated by SRC family kinases and signal to promote further spreading and rigidity-sensing contractions on stiff but not soft substrates^46^. Importantly, loss of rigidity-sensing contractions confers to cancer cells the ability to grow on soft substrates, independently from stable integrin-mediated anchorage to the ECM^47^.

In triple-negative breast cancers (TBNCs), which are very aggressive tumors characterized by the lack of ER, progesterone receptor (PR), and HER2, high expression and reciprocal compensation between β1 integrin and EGFR signaling synergize to support tumor progression and metastasis^48^. Ectopic expression of the secreted ECM protein TINAGL1 (tubulointerstitial nephritis antigen-like 1) suppresses TNBC progression by binding and inhibiting the signaling of both β1 integrin and EGFR^48^. On the surface of breast, pancreatic, and lung cancer cells, galectin-3 binds and elicits the anchorage-independent clustering of αvβ3 integrin along with the downstream activation of KRAS, a driver of resistance to anti-EGFR therapy^49^. Galectin-3–induced αvβ3 integrin clustering drives KRAS addiction in tumor cells, and galectin-3 inhibition represents an actionable target to treat KRAS G12D-bearing cancers^50^.

Interestingly, the laminin receptor β4 integrin binds to several TKRs, including EGF-R, HER2 (also known as ERBB2), MET, and RON, sustaining their signaling pathways^51–54^. Upon ligand stimulation, these TKRs activate integrin-associated SRC-family kinases (SFKs) promoting the phosphorylation of the β4 cytotail and TKR-bound interactors by SFKs. These multiple phosphorylations create an amplified docking platform potentiating TKR downstream pathways favoring hemidesmosome disassembly and cancer cell invasion^55,56^. These findings suggest the possible benefit of the combined use of anti-cancer agents able to interfere with integrin signaling function in combination with the impairment of their adhesive properties.

### Integrins and cancer cell metabolism

**Figure 2.**
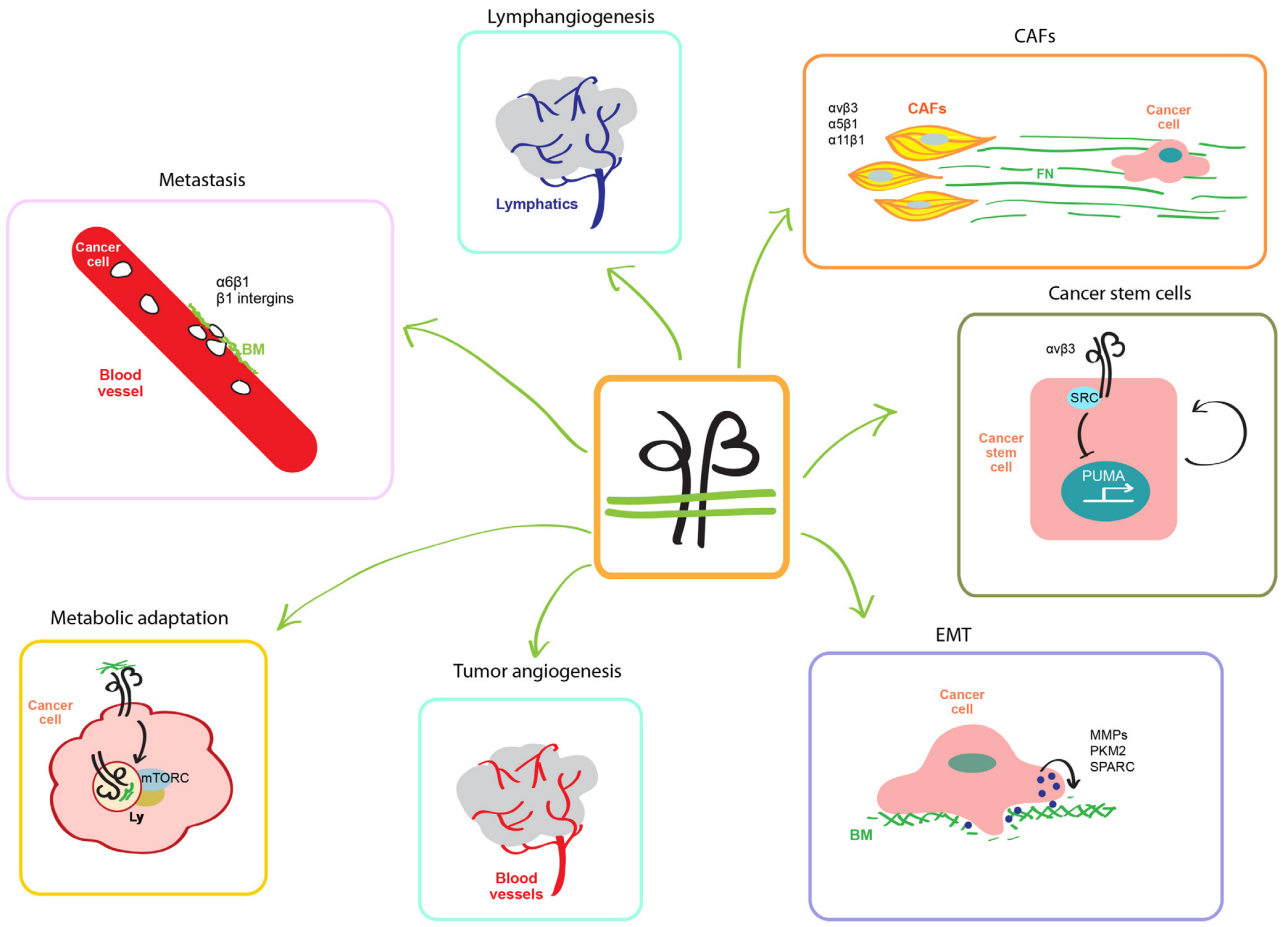
Integrins are involved in several steps of cancer progression. Integrins and stiff extracellular matrix regulate the metabolic behavior of cancer cell and mTORC activation on lysosomes (Ly). Integrins promote the formation of new blood and lymphatic vessels that support tumor growth, invasion, and metastatic dissemination. During epithelial-to-mesenchymal transition (EMT), integrins sustain the migratory phenotype of cancer cells, also characterized by an increased secretion of matrix metalloproteinases (MMPs), the M2 isoform of pyruvate kinase (PKM2), and the matricellular protein SPARC (secreted protein acidic and rich in cysteine). In breast cancer, αvβ3 integrins induce the transcription of the proapoptotic gene PUMA (p53-upregulated modulator of apoptosis), thus supporting tumor stemness. In the tumor microenvironment, CAFs produce and polymerize via α5β1 high amounts of aligned fibronectin (FN) fibers along which cancer cells migrate directionally in an αvβ3-dependent manner. Furthermore, α11β1 integrin– and PDGFR-β–expressing CAFs promote breast cancer progression. Integrin-mediated interactions with laminin of blood vessel basal membrane (BM) also promote cancer cell dissemination and seeding in distant organs.

Recent evidence sheds further light on the critical role played by integrin and ECM stiffness in shaping metabolic behaviors of cancer cells^57,58^. Mammalian target of rapamycin (mTOR) and PI3K signaling allow tumor cells to grow in a nutrient-deprived environment and to overcome anoikis in anchorage-independent conditions^59^. Although PI3K/mTOR inhibition results in apoptosis of ECM-detached tumor cells, ECM-adherent tumor cells respond to nutrient deprivation by inducing the expression of pro-survival and anti-apoptotic proteins, such as BCL2, EGFR, and insulin-like growth factor 1 receptor (IGF1R)^60^. Starved mammary epithelial cells increase β4 integrin expression and endocytosis of β4 integrin-bound laminin, whose degradation in lysosomes increases the availability of amino acids and enhances mTORC1 signaling, thus preventing cell death (Figure 2)^61^. Because β4 integrin overexpression is a feature of several human cancers^62^, it will be interesting to investigate whether β4 integrin-dependent laminin internalization represents a key amino acid source in tumors.

Activated α5β1 integrins move from peripheral nascent adhesions to central fibrillary adhesions in a tensin-dependent manner. In ovarian cancer cells, Arf4-driven internalization from fibrillary adhesions of ligand-engaged α5β1 integrins triggers their endosomal transport to lysosomes, promoting mTOR activation^63^. In contrast, the formation of fibrillary adhesions in fibroblasts is prevented by the energy sensor 5′-prime-AMP-activated protein kinase (AMPK), which downregulates the expression of tensin^64,65^. AMPK is activated by the cellular AMP/ATP ratio increase that occurs in conditions of nutrient depletion. Hence, AMPK and mTOR, by responding to opposite alterations in nutrient availability, are reciprocally regulated and control tensin-coupled α5β1 integrin-driven FN fibrillogenesis. The AMPK-regulated turnover of the FN matrix may be relevant for tumor–CAF crosstalk in that CAF-derived FN could be a source of metabolic energy for cancer cells.

In a HER2-driven mouse model of breast cancer, a stiff ECM induces insulin receptor (IR) recruitment to focal adhesions, thus allowing the formation of IR/integrin complexes and preventing IR lysosomal degradation^66^. Double β1/β3 integrin genetic ablation in this breast cancer model results in defective IR/AKT/mTORC1 signaling and inhibition of both tumor onset and metastatic dissemination to lungs^66^. On a related note, within the proneural and classic subgroups of patients with GBM, a subpopulation exists in which high αvβ3 integrin levels activate a PAK4-mediated YAP/TAZ signaling pathway that in turn enhances the expression of the insulin-regulated and neuron-specific glucose transporter GLUT3. High αvβ3 integrin/GLUT3-addicted GBM cells survive in a glucose-deprived milieu and are resistant to conventional therapies^67^.

Finally, it is becoming apparent that the availability of specific nutrients in different districts of the organism may draw a heterogeneous landscape of area-specific environmental pressures that are crucial for the selection of metastatic sites by circulating cancer cells. For example, the abundance of pyruvate in lung interstitial fluids supports the production of α-ketoglutarate that in breast cancer cells increases the activity of the collagen prolyl-4-hydroxylase enzyme. As a result, pyruvate promotes collagen crosslinking and ECM remodeling by breast cancer cells in this metastatic niche, thus specifically improving their survival in lungs^68^.

## Integrin in the context of cancer progression and therapy

### Integrins and tumor blood vessels

Vascular endothelial growth factor (VEGF)^69^ and integrins^3^ drive the formation of nutrient-providing blood vessels that support tumor growth and progression. Angiogenesis depends on dynamic cycles of EC adhesion to deadhesion from the ECM that crucially depends on the endosomal traffic of integrins^70^. In this regard, the Arg-Gly-Asp (RGD) motif-containing and integrin-binding epidermal growth factor-like protein 7 (EGFL7) is emerging as a novel important regulator of tumor angiogenesis (Figure 2)^71^. For instance, ECs of glioblastoma (GBM) blood vessels express high levels of EGFL7 that binds, impairs the endocytosis, and promotes the accumulation of αvβ3 (and α5β1) integrin on the surface of ECs^72^. In GBM, endothelial EGFL7–integrin signaling promotes the formation of new blood vessels and therapeutic EGFL7 blockade synergizes with anti-VEGF to more effectively impair tumor angiogenesis and growth^72^.

The stiffening of the ECM of tumor microenvironment (TME) also promotes angiogenesis^73^, likely via a mechano-signaling network triggered by Agrin, a heparan sulfate proteoglycan that both cancer cells^22^ and ECs^74^ secrete. Agrin is well known for its ability to bind to low-density lipoprotein receptor-related protein 4 (LRP4) receptor that, activating muscle-specific kinase (MUSK), triggers the aggregation of acetylcholine receptors and the formation of neuromuscular junctions^75^. During cancer progression, Agrin-bound LRP4 associates instead with β1 integrins and elicits FAK signaling that in turn fosters tumor angiogenesis and growth by stabilizing VEGFR-2 protein levels in ECs^74^.

Recently, Wong *et al*. showed how tumor growth also depends on novel integrin-dependent non-canonical function of pericytes that act as paracrine sources of tumor trophic factors^76^. Indeed, in different human cancers, the simple lack of β3 integrin in blood vessel mural cells correlates with a larger tumor size and clinically more aggressive phenotype^76^. Mechanistically, the absence of β3 integrin in pericytes, though not altering blood vessel number and function, upregulates the FAK-MET-AKT-NFKB–driven production of the cytokine CCL2 that in turn sustains tumor growth in a CCR2 receptor–dependent manner^76^.

### Integrins and tumor progression

During tumor progression, transcription factors of the SNAIL, TWIST, and ZEB gene families give rise to the EMT transcriptional program, which is characterized by the detachment from the epithelial basement membrane (BM) and the acquisition of a β1/β3 integrin and FN-driven migratory phenotype, endowing cancer cells with increased invasive ability and resistance to therapies^77^. During EMT, the increased transcription of genes encoding for matrix metalloproteinases (MMPs) facilitates the degradation of BM by cancer cells^77^. The non-canonically secreted M2 isozyme of pyruvate kinase (PKM2) accumulates in the extracellular fluids of several human cancers and promotes cancer progression^78^. In lung cancer cells, secreted PKM2 was found to bind β1 integrins and elicit the activation of a FAK/SRC/ERK pathway that facilitates metastasis, at least in part by increasing MMP-9 transcription^79^.

EMT is also supported by matricellular proteins, such as secreted protein acidic and rich in cysteine (SPARC) that, in addition to binding and activating integrin signaling, crosslinks and increases the stiffness of TME ECM (Figure 2)^80^. In addition, SPARC drives cancer EMT by modulating TME immune cells. Indeed, in high-grade breast cancers containing high amounts of SPARC, the deposition of abundant collagen bundles induces the COX-2–dependent expression of granulocyte-macrophage colony-stimulating factor (GM-CSF) and interleukin-6 (IL-6) in tumor cells attracting CD33^+^ myeloid-derived suppressor cells responsible for cancer cell EMT and chemoresistance^81^. In addition, EMT promotes the generation of self-renewing cancer stem cells (CSCs) that actively sustain tumor development and growth through molecular mechanisms that are under investigation^82^. In breast CSCs, αvβ3 integrin-activated SRC kinase signals to suppress via SLUG (also known as SNAI2) the transcription of the proapoptotic gene p53-upregulated modulator of apoptosis (PUMA), thus supporting tumor stemness (Figure 2)^83^. Significantly, pharmacological αvβ3/SRC blockade fosters breast CSC apoptosis and lessens pulmonary metastasis by re-establishing PUMA expression^83^.

### Integrins and tumor microenvironment

Adhesive and humoral interactions with TME cells (for example, fibroblasts, blood and lymphatic ECs, and immune cells) play key roles in supporting cancer metastasis colonization of distant sites throughout the body. First identified as “myofibroblasts” in healing wounds^84^ and then in breast cancer^85^, CAFs and their role in tumor progression and invasion started being appreciated and investigated in only the last two decades^86^. CAFs release diffusible factors that are not sufficient to elicit cancer cell invasion, which instead crucially relies on their ability to remodel the ECM (Figure 2)^87,88^. In the extracellular microenvironment, α5β1 integrin binds dimeric soluble FN and promotes its polymerization in a matrix that functions as a scaffold driving type I collagen fibrillogenesis^89^. *In vitro*, CAFs isolated from human tumors produce and polymerize—via coordinated α5β1/αvβ3 integrin-binding and platelet-derived growth factor receptor alpha (PDGFRα)-mediated actomyosin contractility—an abundant anisotropically oriented FN fibrillar network along which cancer cells migrate directionally in an αv integrin–dependent manner^87,88^. Furthermore, aligned FN fibrils characterize invasion sites in human prostatic ductal adenocarcinoma and PDAC^88^. Analyses of the preclinical MMTV-PyMT (mouse mammary tumor virus polyoma middle tumor antigen) model and human samples unveiled the crucial role played by a subset of CAFs expressing the collagen-binding integrin α11β1 and PDGFRβ in breast cancer progression^90^. Mechanistically, the PDGF-BB–dependent interaction of α11β1 integrin with PDGFRβ activates, through JNK, the production by CAFs of the matricellular protein tenascin C^80^, which in turn promotes breast cancer cell invasion^90^.

Different cancer types may employ diverse (circulation or lymphatic) routes for metastatic dissemination, depending on the embryonic derivation and the anatomy of the tissue of origin. Moreover, studies in mouse models have shown that cancer cells first can metastasize to lymph nodes and next enter the circulation by intravasating through lymph node blood vessels^91,92^. Accordingly, lymphangiogenesis frequently precedes metastatic invasion of solid tumor-draining lymph nodes (Figure 2)^93^. Lymphatic ECs of cancer-associated lymph nodes *de novo* express the αIIb integrin subunit that, together with β3, gives rise to the main fibrinogen receptor^94^. Indeed, αIIbβ3-expressing lymphatic ECs adhere to fibrinogen *in vitro* and fibrinogen associates with αIIbβ3^+^ lymphatic ECs in tumor-draining lymph nodes *in vivo*, pointing to a role of αIIbβ3 integrin in cancer-linked lymphatic remodeling^94^. Concerning the mechanisms supporting lymphatic invasion, α6β4 integrin-expressing macrophages in TBNCs adhere to BM laminin 5 of the lymphovasculature and release TGFβ1, thus eliciting RhoA-dependent lymphatic EC contraction that favors tumor intravasation^95^.

### Integrins and metastatic dissemination

Integrins are involved in each of the major steps of solid tumor metastatic progression from cancer cell breaching the BM, entering into blood or lymphatic vessels (intravasation), anchorage-independent survival in the circulation, exit from vasculature (extravasation), and colonization of distant organs. At least two independent studies^96,97^ have shown how metastatic cancer cell dissemination and seeding in distant organs, such as brain, lung, and liver, can rely on the integrin-mediated interaction with BM laminin on the abluminal side of blood vessel ECs, a tumor survival and progression strategy that requires blood vessel co-option (Figure 2)^98^. Indeed, upon extravasation, metastasis-initiating cancer cells colonize different organs by competing with pericytes for the perivascular niche and adopting a common pathway in which L1CAM supports BM-driven β1 integrin activation and ILK/YAP signaling^96^. Similarly, acute lymphoblastic leukemia (ALL) metastatization to central nervous system (CNS) leptomeninges depends on the PI3Kδ-dependent expression of α6β1 integrin that supports ALL cell extravasation and migration along laminin of BM that coats the external surface of meningeal blood vessels^97^. Pharmacological inhibition of PI3Kδ, which downregulates α6 integrin expression, or α6 integrin direct blockade significantly impairs ALL spreading along CNS^97^ as well as resistance to chemotherapy in mouse models of B-cell ALL (B-ALL)^99^. These data, along with the observation that high expression levels of α6 integrin at the time of diagnosis in patients with high-risk B-ALL predict resistance to standard-of-care therapy, suggest that α6 integrin may represent a novel therapeutic target in ALL. Similarly, in the perivascular niche, αvβ3 and α4β1 integrin-mediated interaction of bone marrow–disseminated breast cancer cells with EC-derived ligands (that is, von Willebrand factor and VCAM-1 respectively) protects them from chemotherapy, whose cytotoxic and anti-metastatic effects can be unleashed by the co-administration of αvβ3 or β1 integrin antagonists^100^. Likewise, inhibition of integrin function in TME cells can be effective in improving the efficacy of chemotherapy, at least in some cancer types such as PDAC, which is characterized by the abundant accumulation of stromal ECM. Samples from patients with PDAC display significantly high levels of α5 integrin expression in stromal cells that inversely correlate with overall survival. ITGA5 gene silencing in pancreatic stellate cells and functional inhibition of α5 integrin with a peptidomimetic compound strongly reduced desmoplasia and significantly increase the therapeutic efficacy of gemcitabine in co-injection in patient-derived xenograft tumor models of PDAC^101^.

Investigations of *in vitro* and *in vivo* models of head and neck squamous cell carcinoma (HNSCC) revealed that β1 integrins can favor the activation of the classic non-homologous end joining (NHEJ) mechanism of repair of DNA double-strand breaks (DSBs) induced by radiotherapy^102^. β1 integrins indeed connect poly-ADP-ribose polymerase 1/2 (PARP1/2) to DSB sensor proteins Ku-70/80, but not to DNA single-strand break (SSB) sensors, through still-unknown mechanisms. Of note, even if β1 integrin blockade increases HNSCC radiosensitivity, the additive effect observed after the simultaneous inhibition of PARP1/2 suggests that β1 integrins promote the activation of some but not all mechanisms of DNA repair. Interestingly, it was recently reported that simultaneous but not single inhibition of αv and β1 integrins effectively overcomes the radioresistance, prevents the metastatic escape, and enhances the long-term survival of sarcoma and melanoma mouse models^103^. In this regard, it may be worth investigating whether αv and β1 integrins signal to promote the activity of different DNA repair machineries.

### Targeting integrins in anti-cancer therapy

Integrins were considered attractive pharmacological targets for drugs aimed at hampering several key processes in cancer progression, such as cell proliferation, survival, and migration. However, despite initial encouraging results in preclinical studies, monoclonal antibodies, antagonist peptides, and small molecules used as inhibitors of integrin functions were largely unsuccessful in clinical trials and so far none of them has been recognized as an effective anti-cancer drug (see Table 1 for examples of integrin-inhibiting agents)^104^. Targeting integrins to improve the delivery of anti-tumor agents or to image cancer lesions represents a novel and promising approach. Cyclic RGD-based nanocarriers coupled to anti-cancer agents have been used successfully to target αvβ3 integrin-overexpressing angiogenic ECs or cancer cells^105^. These carriers, being internalized via receptor-mediated endocytosis, allow an efficient intracellular delivery of drugs and their efficacy has been further improved when exploited in dual targeting. The simultaneous delivery of peptides directed against αvβ3 integrin and P-selectin^106^ or CD44^107^ displayed increased anti-angiogenic and anti-cancer efficacy when compared with single targeting strategies. Interestingly, an αvβ6 integrin-specific peptide was coupled to a photosensitizer and used to increase the efficacy of photodynamic therapy in a lung mouse model. Local activation by light irradiation of the compound induces the production of reactive oxygen species, necrotic cancer cell death, and dendritic cell– and CD8^+^ T cell–mediated response^108^. Tracers targeting αvβ3, α5β1, and αvβ6 have also been developed and exploited for *in vivo* tumor imaging by magnetic resonance imaging, positron emission tomography, and computer tomography^109–111^. These techniques are potentially relevant for the initial diagnosis, staging, and monitoring of the disease as well as intraoperative visualization of tumor margins to guarantee their complete resection^112^.

**Table 1.** Examples of integrin-inhibiting agents used in cancer therapy.

Integrin-inhibiting agent		Target	Clinical phase	Type of cancer	Effect	Outcome	References
**Monoclonal** **antibodies**	Intetumumab	αv	I and II	Melanoma, sarcoma, prostate, and colorectal cancer	Inhibits the contact between extracellular matrix (ECM) and tumor cells and inhibits angiogenesis	Improved overall survival with high doses or no benefits	118,119
	Abituzumab	αv	I and II	Melanoma, sarcoma, prostate, and colorectal cancer	Inhibits the contact between ECM and tumor cells and inhibits angiogenesis	No benefits or trials ongoing	120
	Vitaxin	αvβ3	I and II	Metastatic cancers	Inhibits cell adhesion, blocks apoptotic pathway, and upregulates matrix degradation inhibitors	Short half-life and inefficient interaction. No tumor regression	121,122
	Etaracizumab	αvβ3	I and II	Melanoma	Inhibits the contact between ECM and tumor cells and inhibits angiogenesis	Good tolerability and no anti-angiogenic or immunomodulatory effects	123
	Volociximab	α5β1	I and II	Ovarian, pancreatic and renal cancer, melanoma, and non-small-cell lung cancer	Inhibits interaction with ECM, induces apoptosis in cancer cells, and inhibits angiogenesis	Good tolerability, anti-angiogenic effects, or no benefits	124
**Peptides**	Cilengitide	αvβ3 αvβ5	I, II, and III	Multiple cancer, including glioblastoma	Compete with ligand binding and inhibit angiogenesis and cancer cell invasion	No therapeutic benefits	125–131
	ATN-161	α5β1	I and II	Lung, colon	Anti-tumor, anti-adhesive, and anti- metastatic activities and inhibits angiogenesis	No therapeutic benefits	132
**Small-molecule** **inhibitors**	GIPG0187	Arg-Gly-Asp (RGD)-binding integrins	I	Advanced cancers	RGD antagonist	Ongoing, reduced liver metastasis	133

Because integrins have a crucial role in cancer development, the failure of numerous clinical studies inhibiting their functions has been disappointing and opens new questions that still lack conclusive answers. Although many integrin αβ heterodimers have been implicated in tumor development, only a few of them have been exploited as therapeutic targets in clinical trials. Moreover, functional redundancy of integrins suggests the possible efficacy of therapies simultaneously targeting multiple integrin heterodimers. Conventional integrin-inhibiting anti-cancer drugs have been conceived for the ability to impair ligand binding. However, when bound to high-affinity compounds, integrins could still promote intracellular signals and survival of cells in ECM-detached conditions. Identifying molecules capable of interfering with the recruitment of cytoplasmic interactors, such as talins, kindlins, ILK, or FAK, may represent an alternative rationale for the selection of novel pharmacological classes of integrin inhibitors.

Although most integrin inhibitors were used mainly for single-agent anti-angiogenic therapies, other components of TME, other than blood vessels, such as infiltrating immunosuppressive cells, whose inhibition might improve cancer-directed immune response^113^, and CAFs could represent novel targets for integrin-based therapies. For example, by impairing the adhesion of monocytes to the vascular endothelium and their extravasation into tumor tissues, α4β1 integrin antagonists were found to hamper macrophage colonization of tumors and angiogenesis^114^. In addition, targeting αv and β1 integrin subunits hinders ECM deposition and remodeling by CAFs, ultimately lessening cancer aggressiveness^115^. The peculiar expression of αvβ6 integrin, highly upregulated in colon, lung, breast, cervix, ovaries/fallopian tube, pancreas, and head and neck solid tumors, while physiologically absent in adult epithelial tissues, renders this receptor an extremely attractive target for immunotherapeutic strategies such as chimeric antigen receptor T-cell adoptive immunotherapy. Moreover, the latency-associated peptide (LAP), which maintains TGFβ in its inactive form, is a major ligand of αvβ6 integrin. Because the binding to αvβ6 integrin induces LAP conformational changes releasing active TGFβ, which signals to suppress T-helper 2 cells and activate MMPs, functional blocking of this integrin may hamper tumor progression by improving immunotherapy and decreasing matrix degradation and cell invasion^116,117^. Finally, a further strategy to improve the impact of integrin-targeting therapies is represented by the identification of new suitable biomarkers to be employed for successfully selecting patients who would benefit more from this type of pharmacological treatment.

## Summary

Several recent studies have provided evidence regarding the involvement of integrins and their upstream modulators and downstream effectors in the development, progression, and resistance to standard-of-care therapies in different cancer types. In addition to affecting cell adhesion, motility, invasion, and metastatic dissemination, integrins were reported to have an impact on cancer cell stemness, anchorage-independent growth, anoikis, and metabolism. The disappointing outcomes of the numerous clinical trials employing integrin inhibitors as single anti-cancer agents increase the possibility that these therapies are more effectively exploited in combination. Indeed, likely because of the unique ability of integrins to simultaneously transduce mechanical and biochemical signal and functionally interact with several other receptor systems (for example, growth factor and G protein–coupled receptors) and metabolic and signaling pathways (for example, DNA damage repair), their pharmacological inhibition may be particularly effective in rescuing cancer resistance to chemotherapy, targeted therapy, and radiotherapy. Further comprehensive characterization of the molecular and cellular mechanisms by which integrins control cancer cell behavior will allow a more effective set of strategies to exploit integrin inhibition in anti-cancer therapies.
